# Effects of dual-task paradigm on the injury potential during landing among individuals with chronic ankle instability

**DOI:** 10.3389/fphys.2024.1473844

**Published:** 2024-11-28

**Authors:** Cheng Zhong, Xin Luo, He Gao, Teng Zhang, Xiaoxue Zhu, Xueke Huang, Peixin Shen

**Affiliations:** ^1^ Graduate School, Shandong Sport University, Jinan, China; ^2^ College of Sports and health, Shandong Sport University, Jinan, China

**Keywords:** ankle sprain, dual-task paradigm, drop-landing, ankle inversion angle, ankle inversion angular velocity

## Abstract

**Purpose:**

Chronic ankle instability (CAI) causes maladaptive neuroplastic changes in the central nervous system, which may lead to high injury potential under dual-task conditions. This study aims to explore the effects of dual-task paradigm on the injury potential during landing among individuals with CAI.

**Methods:**

Twenty participants with CAI (4 female and 16 male, 12 were affected with their right limbs and 8 were affected with their left limbs, 20.4 ± 1.7 years, 176.9 ± 5.0 cm, and 72.0 ± 11.1 kg) and eighteen without CAI (6 female and 12 male, 20.2 ± 1.5 years, 173.5 ± 7.0 cm, and 70.3 ± 10.8 kg) were recruited. They drop-landed on a trap-door device, with their affected or matched limbs on a flippable platform, under single- (drop-landing only) and dual-task (drop-landing while subtracting of serial threes) conditions. A twelve-camera motion capture system was used to capture the kinematic data. Two-way ANOVA with mixed design (CAI vs non-CAI groups by single-vs dual-task conditions) was used to analyze the data.

**Results:**

Significant group-by-condition interactions were detected in the ankle inversion angle (*P* = 0.040, *η* ^2^_*p*_ = 0.012) and ankle inversion angular velocity (*P* = 0.038, *η* ^2^_*p*_ = 0.114). Both indicators decreased among individuals without CAI from single-to dual-task conditions, while remained unchanged among those with CAI; and they were higher among individuals with CAI under both single- and dual-task conditions, compared to those without CAI.

**Conclusion:**

Individuals with CAI have a reduced ability to limit ankle inversion compared to those without CAI. Under dual-task conditions, individuals without CAI limited their ankle inversion, while those with CAI did not. Drop-landing, especially under dual-task conditions, poses a high risk of excessive ankle inversion for individuals with CAI.

## 1 Introduction

Ankle sprains are one of the most common sports injuries, accounting for 10%–20% of all sports-related injuries (Fong et al., 2007) and up to 70%–80% among college students with sports experience (Doherty et al., 2014). In the Netherlands, approximately 440,000 ankle sprains occur annually (Kemler et al., 2015), while in the United Kingdom, they constitute 3%–5% of all emergency department visits, resulting in 1–1.5 million cases per year (Gribble et al., 2016b). Acute ankle sprains can lead to repeated injuries and the development of chronic ankle instability (CAI), with a prevalence of about 20%–30% (Konradsen et al., 2002; Herzog et al., 2019). CAI is characterized by pain, instability, recurrent injuries, and persistent dysfunction (Gribble, 2019), significantly impacting an individual’s physical activity levels (Hubbard-Turner and Turner, 2015).

Landing from a height is a common scenario leading to ankle sprains (Doherty et al., 2014; Ardakani et al., 2019). During landing, the foot and ankle complex absorb the impact force from the ground, which can be 2–5 times the body weight (Xu et al., 2024b; Xu et al., 2023). This force is primarily transmitted through the medial aspect of the ankle, may cause sudden and substantial inversion of the ankle joint, which can lead to ankle sprains (Koshino et al., 2017). Larger ankle inversion angles and angular velocities during landing are associated with an increased potential for ankle sprains (Xu et al., 2024a; Zhang et al., 2024). Individuals with CAI have greater ankle inversion angles and angular velocities during landing compared to those without CAI (Simpson et al., 2022; Terrier et al., 2014). Excessive ankle inversion increases the distance between the talus and fibula, stretching the ligaments connecting these bones (Fong et al., 2012). When the ligament is stretched beyond its maximum bearing capacity, it can lead to ligament tears (Medina McKeon and Hoch, 2019).

CAI, a neurophysiological disorder characterized by maladaptive neuroplastic changes in the central nervous system (CNS), may increase potential injury under dual-task conditions. Individuals with CAI often exhibit reduced activation of the dorsal anterior cingulate cortex (Shen et al., 2022), a critical region for integrating cognitive resources (Bush et al., 2000). This reduction in cognitive resources can impair motor performance and elevate injury risk in environments requiring additional cognitive demands, such as dual-task conditions (Rosen et al., 2021; Choi et al., 2023). For example, a volleyball player with CAI aiming to execute a powerful smash must jump as high as possible and extend their upper limb. This constitutes a motor task. Simultaneously, they must consider the optimal landing spot for the ball based on the opposing defender’s position and condition, representing a cognitive task. Together, these actions embody a dual-task condition. Individuals with CAI, who have a limited total capacity of potential cognitive resources, experience reduced allocation of these resources to both cognitive and motor tasks. Consequently, their performance on either task may suffer, potentially leading to tactical errors or unintentional injuries.

There is ongoing controversy regarding the performance of individuals with and without CAI during dual-task conditions, as well as the potential impact of dual-task paradigms on injury risk in individuals with CAI. Some researchers report an increase in injury potential among individuals with FAI when transitioning from single-task to dual-task conditions, attributing this to impaired feedforward and feedback mechanisms of motor control within the CNS (Tavakoli et al., 2016). Conversely, others have observed enhanced postural stability among CAI individuals under dual-task conditions, which they attribute to increased conscious control over body movement due to fear of anticipated pain or reinjury (Shiravi et al., 2017). Moreover, it remains uncertain whether CAI individuals are more susceptible to the effects of dual-task paradigms compared to those without CAI. Some studies have indicated that athletes with FAI exhibit poorer postural stability than healthy controls under dual-task conditions, potentially increasing their injury risk (Rahnama et al., 2010). However, other studies have reported no differences in postural stability between CAI and non-CAI individuals under dual-task conditions (Choi et al., 2023).

Investigating the effects of dual-task paradigm on the injury potential among individuals with CAI may enhancing our understanding on whether the maladaptation of CNS affect the ankle sprain recurrence, and even the tactical arrangements of when and how long the players with CAI would be in the game. Therefore, we hypothesized that: 1) compared to individuals without CAI, those with CAI have higher injury potential, reflected by greater ankle inversion angle and angular velocity. 2) compared to single-task conditions, the injury potential increased among individuals with and without CAI under dual-task conditions.

## 2 Methods

### 2.1 Participants

Sample size calculations were conducted using G*Power 3.1 software (University of Düsseldorf, Düsseldorf, Germany). Prior to the formal study, a pilot study was performed with six participants with CAI and another six participants without CAI. The ankle inversion angle and ankle inversion angular velocity were used as outcome measures to estimate the sample size. The effect sizes (*η* ^2^_*p*_) for the group comparison (CAI vs. non-CAI) by condition (single-task vs. dual-task) were 0.064 and 0.087, respectively. Based on these calculations, a total of at least 34 participants (17 in each group) were required to achieve a statistical significance level of 0.05 and a statistical power of 0.80.

Participants were recruited in a local university from April to June 2023 through distributing posters and leaflets. Following the guidelines of the International Ankle Consortium (Gribble et al., 2016a), the inclusion criteria for participants with CAI were: 1) at least one severe ankle sprain a year prior to the recruitment, causing pain, swelling, and other inflammatory symptoms, inhibiting normal participation in daily activities for more than 1 day; 2) aged 18–24 years (Ardakani et al., 2019); 3) at least two episodes of ankle “giving way” in the past 6 months; 4) persistent sense of ankle instability and impaired ability of daily activities and 5) with a score <24 of the Cumberland Ankle Instability Tool (CAIT) (Hiller et al., 2006). Inclusion criteria for participants without CAI were: 1) no previous ankle sprain/injury and no episodes of ankle “giving way” or feeling of ankle instability and 2) CAIT score >28. Exclusion criteria for all participants were: 1) self-reported history of lower limb fractures or surgery; 2) experienced acute injuries such as sprains in the lower limbs 3 months prior to the recruitment; 3) bilateral chronic ankle instability. All participants in this study were regular college students, both with and without CAI, who attended physical education classes three times a week for 45 min per session. They were not sedentary and were in good physical condition. After the assessment, 38 participants met the inclusion criteria, of whom 20 with CAI (4 female and 16 male, 12 were affected with their right limbs and 8 were affected with their left limbs, 20.4 ± 1.7 years, 176.9 ± 5.0 cm, and 72.0 ± 11.1 kg) and 18 without CAI (6 female and 12 male, 20.2 ± 1.5 years, 173.5 ± 7.0 cm, and 70.3 ± 10.8 kg) were recruited. This study was approved by the Institutional Review Board of Shandong Sport University (Approval Number: 2023014) and was in accordance with the Declaration of Helsinki, and all participants signed informed consent forms.

### 2.2 Protocols

**FIGURE 1:**
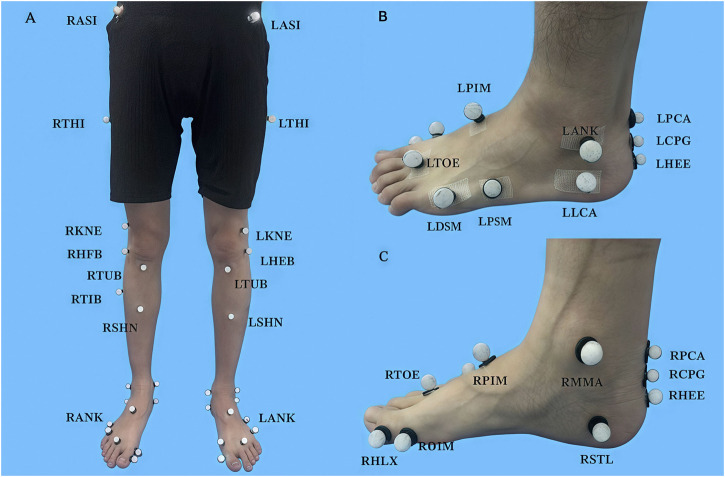
Oxford Foot Ankle Model **(A)** Front view of full markers. **(B)** Lateral view of foot markers. **(C)** Medial view of foot markers.

Participants wore uniformed tight shorts and T-shirts, and 36 markers were adhered to their lower limbs, following the protocol of the Oxford Foot Ankle Model (McCahill et al., 2008) (Figure 1). The test limb in the CAI group were the affected limb, and the test limb in the non-CAI group were matched based on the ratio of the right and left affected limb in the CAI group, from which the number of right limbs tested in the non-CAI group was calculated to be: 12/20*18 = 10.8. i.e., 11 participants in the non-CAI group tested the right side, and 7 participants tested the left side. The non-CAI participants were randomized to the computerized array, which was used to determine the side of limb to be tested. Before formal tests, participants familiarized themselves with the procedure by conducting at least 3 drop-landing trials. Then, they conducted formal drop-landing tests under single- and dual-task conditions in a randomized order.

#### 2.2.1 Drop-landing tests

**FIGURE 2:**
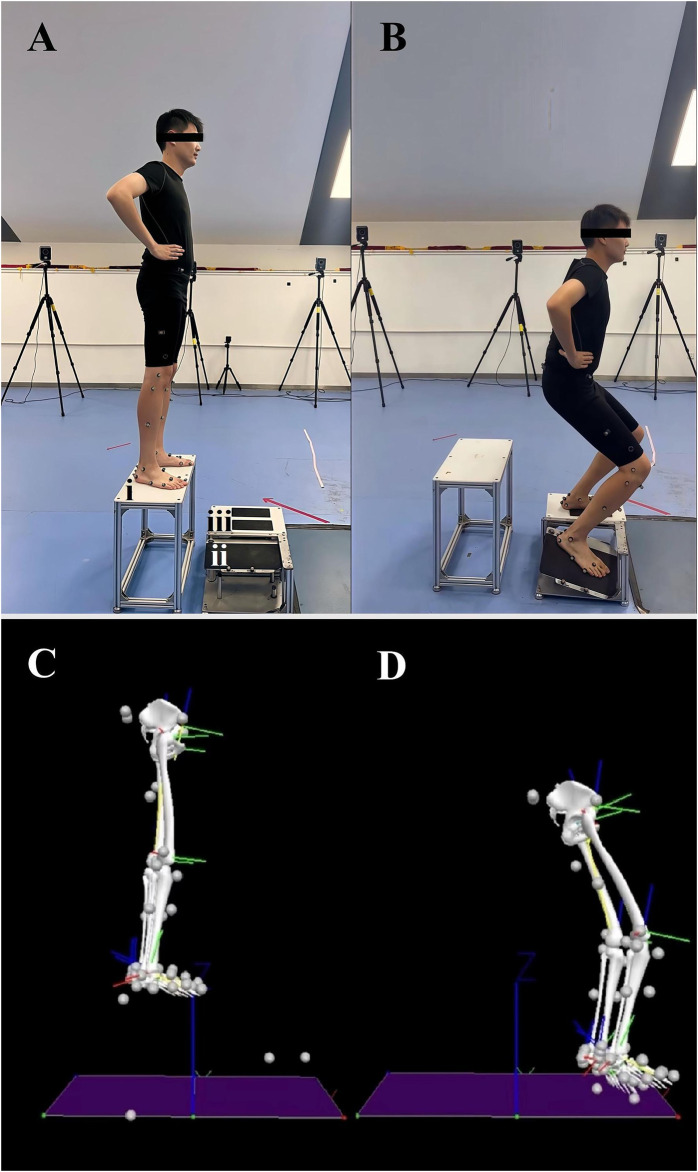
Illustration of drop-landing tests **(A)** Body position before drop-landing. i, take-off platform; ii. flippable platform; iii, supporting platform; **(B)** Body position after drop-landing; **(C)** A free body diagram of body position before drop-landing; **(D)** A free body diagram of body position after drop-landing.

Participants drop-landed from a height of 30 cm (Mackala et al., 2020) to a custom-made trap-door device consisting of three platforms, namely, take-off, flippable, and supporting platforms (Figure 2A). The height of 30 cm for landing has been proven safe and is widely applied in the literature (Shibata et al., 2023; Mokhtarzadeh et al., 2017; Watanabe et al., 2022; Li et al., 2018; Huang et al., 2024; Lim et al., 2020). The surface of the flippable platform would be flipped when subjected to a force >10 N. A marker was attached to the lateral edge of the flippable platform surface to identify the time point when it flipped. During the drop-landing test, the participants’ kinematic data were recorded by a 12-camera, 3D infrared motion capture system (Vicon Vantage V5, Oxford Metrics Limited, Oxford, United Kingdom), with a frequency of 100 Hz.

Single-task conditions: Participants stood on the take-off platform, with their eyes looking straight ahead and hands on hips, and extended their affected or matched feet forward. They moved their bodies forward away from the take-off platform to minimize upward movement and then landed on the flippable and supporting platform with the affected or matched and the contralateral limbs, respectively (Figure 2). Participants performed three successful trials, defined as participants being able to stabilize their body and maintain the body position for at least 3 seconds after landing.

**FIGURE 3:**
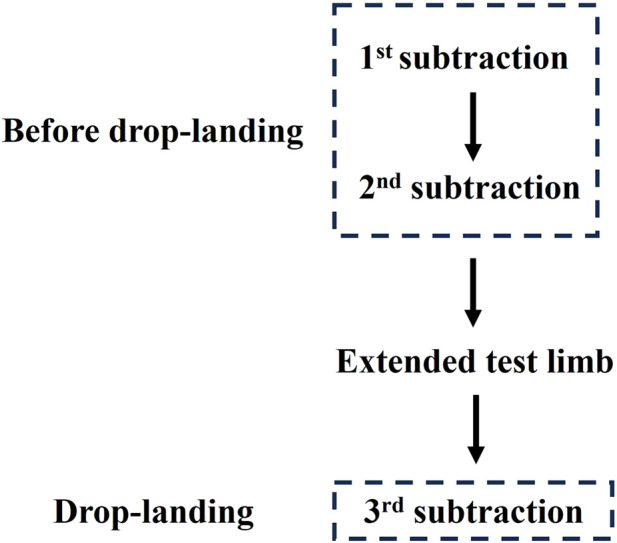
Illustration of drop-landing test under dual-task conditions.

Dual-task conditions: During drop-landing, a subtraction of serial threes from a given three-digit number was performed simultaneously. In each trial, participants subtracted three for three times. Two subtractions were done before drop-landing, and after the second result was given, each participant extended their affected or matched feet forward and performed drop-landing as in single-task conditions, and they gave the result of the third subtraction immediately after landing (Figure 3). To ensure that the subtractions were calculated during landing, the timing of the third subtraction was strictly limited. Prior to the drop-landing test, participants performed consecutive subtractions for three times while sitting in a chair, and the mean time of the three subtractions were recorded as the “sitting subtraction time” (Wrightson et al., 2020). If the time taken for the third subtraction under dual-task conditions during drop-landing exceeded the “sit-subtraction time”, the trial would be deemed unsuccessful. Participants performed three successful trials.

### 2.3 Data reduction

Kinematic data were imported into Visual 3D (V6 professional, C-Motion, Maryland, United States), and low-pass filtered with a cutoff frequency of 12 Hz (Ford et al., 2007). Based on the marker protocol, an 11-segment Oxford foot ankle model was created and embedded in the motion capture files. The data were collected from the time point at landing to 200 ms after landing, defined by the movement of the markers affixed to the lateral edge of the flippable platform surface (Fu et al., 2014). The landing period was chosen because real ankle sprain occurs within it (Fong et al., 2009). The axis of ankle inversion/eversion is defined as the floating axis, which is the common axis perpendicular to both the *Z*-axis in the tibia/fibula coordinate system (the line extending from the tip of the medial malleolus to the tip of the lateral malleolus, directed rightward) and the *Y*-axis in the calcaneus coordinate system (the line aligning with the longitudinal axis of the tibia/fibula in the neutral position, pointing cranially) (Wu et al., 2002).

### 2.4 Variables

The ankle inversion angle was defined as the angle of rotation of the foot coordinate system relative to the tibial coordinate system in the coronal plane during the landing period.

The ankle inversion angular velocity is defined as the peak rate of the change of ankle inversion angle during the landing period, i.e., the peak value of angle increment per unit time.

### 2.5 Statistics

The normality of data distribution was examined using Shapiro-Wilk tests. Mixed model two-way ANOVAs were utilized to compare ankle inversion angle and angular velocity between single- and dual-task conditions among participants with and without CAI. If significant group (CAI vs non-CAI) by condition (single-task vs dual-task) interactions were detected, stratified t-tests with Bonferroni adjustment were used to conduct pairwise comparisons. Partial eta squared (*η* ^2^_*p*_) was used to indicate the effect size of the two-way ANOVA’s interactions and main effects with the thresholds: 0.01∼0.06 for small, 0.06∼0.14 for moderate, and > 0.14 for large effect size (Pierce et al., 2004). Cohen’s *d* was used to indicate the effect size of *post hoc* pairwise comparison with the thresholds: <0.20 for trivial, 0.21∼0.50 for small, 0.51∼0.80 for medium, and >0.81 for large effect size (Cohen et al., 1988). The significance level was set at 0.05.

## 3 Results

**FIGURE 4:**
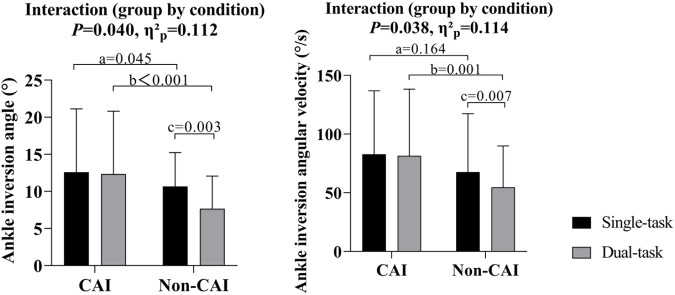
Ankle inversion angle and angular velocity in the CAI and non-CAI populations under single- and dual-task conditions. a. Significant difference between CAI and non-CAI populations under single-task conditions. b. Significant difference between CAI and non-CAI populations under dual-task conditions. c. Significant difference between single- and dual-task conditions in non-CAI populations.

The Shapiro-Wilk test indicated that all the dependent variables were normally distributed (*P* > 0.05). Significant group-by-condition interactions were detected in the ankle inversion angle (*P* = 0.04, *η* ^2^_*p*_ = 0.012) and ankle inversion angular velocity (*P* = 0.038, *η* ^2^_*p*_ = 0.114). *Post hoc* analysis showed that compared to single-task conditions, the ankle inversion angle (*P* = 0.003, *d =* 0.84) and ankle inversion angular velocity (*P* = 0.007, *d =* 0.91) were significantly decreased among individuals without CAI under dual-task conditions, whereas no significant differences were detected among individuals with CAI. Compared to those without CAI, the individuals with CAI had greater inversion angles under single- (*P* = 0.045, *d =* 0.65) and dual-task (*P* < 0.001, *d =* 1.13) conditions, and similarly, they had greater ankle inversion angular velocities under single- (*P* = 0.164, *d =* 0.46) and dual-task (*P =* 0.001, *d =* 1.03) conditions (Figure 4).

## 4 Discussion

To our knowledge, this is the first study to determine the effects of the dual-task paradigm on the injury potential during drop-landing among individuals with and without CAI. The results supported Hypothesis 1, which predicted that individuals with CAI would have a higher injury potential compared to those without CAI, under both single-task and dual-task conditions. This was reflected by a greater ankle inversion angle and angular velocity among individuals with CAI. Conversely, Hypotheses 2 was not supported by the results. Hypothesis 2 proposed that, compared to single-task conditions, the injury potential would be higher among individuals with and without CAI under dual-task conditions, but this was not observed.

The results indicated that individuals with CAI exhibited greater ankle inversion angle and angular velocity compared to those without CAI under both conditions. This finding is supported by a previous study, which showed that individuals with CAI exhibited greater ankle inversion angle after initial contact during landing compared to copers (individuals who suffered ankle sprain but did not develop CAI) and healthy controls (Oh et al., 2024). We propose that the inability to limit excessive ankle inversion during landing is linked to dysfunction in ankle eversion muscles and lateral ankle ligaments. The ankle eversion muscles, especially the peroneal muscles, have been proved to have prolonged reaction time during sudden ankle inversion (Menacho Mde et al., 2010), as well as difficulty generating maximum ankle eversion torque in an inverted position among individuals with CAI (Dong et al., 2024). In the case of sudden ankle inversion, the injured anterior talofibular ligament is unable to limit the separation between the talus and fibula, making it difficult to minimize the ankle inversion angle and angular velocity (Rigby et al., 2015). As a result, individuals with CAI are more likely to be injured than individuals without CAI under both single- and dual-task conditions.

Contrary to Hypothesis 2, a surprising finding was that compared to single-task conditions, ankle inversion angle and angular velocity were lower under dual-task conditions among individuals without CAI. This may because participants adopted different movement strategies under dual-task conditions (McNevin and Wulf, 2002; Lacour et al., 2008). In the capacity sharing theory, when participants engage in dual-tasks, their attention shifts partly from the primary motor task to the supplementary task (in our study, a cognitive task). Consequently, cognitive resources are partially allocated to this additional task, the conscious control over body movements reduced (Lacour et al., 2008). To some extent, automatic and reflexive postural control strategies then take precedence to sustain motor performance (McNevin and Wulf, 2002; Lacour et al., 2008). This shift may result in superior motor performance compared to single-task conditions (Wulf et al., 2004; McNevin and Wulf, 2002; Vuillerme et al., 2000), avoiding the occurrence of injuries. For example, the lower ankle inversion angle and angular velocity observed in our study. A smaller ankle inversion angle indicates that the lateral ankle ligaments experience less strain due to inversion (Yıldız and Yalcın, 2013). Similarly, a lower ankle angular velocity indicates that muscles, such as the peroneal muscles, have adequate reaction time to activate and counteract excessive ankle inversion (Ashton-Miller et al., 1996), which all suggest a potential decrease in the risk of ankle sprains (Koshino et al., 2017).

In contrast to individuals without CAI, those with CAI did not show a decrease in injury potential when transitioning from single to dual-task conditions. The maladaptive neuroplastic changes at both spinal and cortical levels follows musculoskeletal injuries like CAI may be the reasons (Bruce et al., 2020), counteracting the injury potential that should be reduced under dual-task conditions. In the spinal level, the protective mechanism for protecting muscles from excessive strains after injuries, i.e., arthrogenic muscle inhibition (Norte et al., 2022), would affect the ability of voluntary muscle activation. This, in turn, may result in muscle weakness around ankle due to heightened activation of inhibitory interneurons synapsing in the motor neuron pool, thus reducing the efficiency of motoneuron recruitment by the CNS after injuries (Dong et al., 2024). Dong et al. demonstrated that arthrogenic muscle inhibition existed in peroneal muscles among individuals with CAI (Dong et al., 2024), which is the dominating muscles to prevent excessive ankle inversion (Ashton-Miller et al., 1996). The functions of the muscles may be inhibited by the protective mechanism at spinal level, leading to unchanged indicators concerning ankle inversion under dual-task conditions compared to single-task conditions among individuals with CAI. In the cortical level, numerous studies showed that the corticomotor excitability decreased in the projection areas of lower limb muscles around ankle in the M1 among individuals with CAI (Pietrosimone and Gribble, 2012; Terada et al., 2022; Nanbancha et al., 2019; Kosik et al., 2017), making them hard to activate the cortical motor neurons (Pietrosimone et al., 2012; McLeod et al., 2015), then leading to muscle weakness and failure of muscle activation (Pietrosimone et al., 2012). Activities of the secondary sensorimotor cortex and other cortical areas would increase to compensate for the declined cortical excitability in M1 to maintain motor performance (Needle et al., 2017; Bruce et al., 2020), but the cortical compensatory mechanism is vulnerable, which tends to collapse under dual-task conditions, leading to poorer motor performance and higher injury potential (Bruce et al., 2020). Similarly, the concurrent subtraction task may break the compensatory mechanisms at cortical level (Burcal et al., 2019), lead to the inability to confront ankle inversion from single-to dual-task conditions among individuals with CAI during landing.

There are three limitations to this study. First, participants were aware that the flippable platform surface of the trap-door device would flip during landing, so this was an anticipatory condition that may differ from a non-anticipatory condition when a real injury occurs. Second, the gender distribution was not uniform across the two groups in this study, which introduces a potential bias in the results due to gender imbalance. Considering that males and females utilize distinct feedforward control strategies during landing, with females typically activating their knee extensors earlier than males to mitigate the deficit in hip extensor rate tension development (Stearns-Reider and Powers, 2018; Di Giminiani et al., 2020), it is advisable for future research to maintain a balanced gender representation within subgroups to mitigate any potential confounding effects of gender on postural control outcomes. Last, this study did not include direct indicators of muscle activity and ligament status, and it is recommended that more attention be paid to neuromuscular factors in future studies to provide evidence through electromyographic measurements of muscle activity and computer simulation modeling calculations of ligament strain to better assess the potential of ankle injury.

## 5 Conclusion

Individuals with CAI have a reduced ability to limit ankle inversion, inferring increased susceptibility to ankle sprains. Under dual-task conditions, individuals without CAI limited their ankle inversion, while those with CAI did not, inferring a higher injury potential among those with CAI. Drop-landing, especially under dual-task conditions, poses a high risk of excessive ankle inversion for individuals with CAI.

## Data Availability

The raw data supporting the conclusions of this article will be made available by the authors, without undue reservation.

## References

[B1] Ardakani M. K. Wikstrom E. A. Minoonejad H. Rajabi R. Sharifnezhad A. (2019). Hop-stabilization training and landing biomechanics in athletes with chronic ankle instability: a randomized controlled trial. J. Athl. Train. 54, 1296–1303. 10.4085/1062-6050-550-17 31618073 PMC6922560

[B2] Ashton-Miller J. A. Ottaviani R. A. Hutchinson C. Wojtys E. M. (1996). What best protects the inverted weightbearing ankle against further inversion? Evertor muscle strength compares favorably with shoe height, athletic tape, and three orthoses. Am. J. Sports Med. 24, 800–809. 10.1177/036354659602400616 8947403

[B3] Bruce A. S. Howard J. S. H V. A. N. W. Mcbride J. M. Needle A. R. (2020). The effects of transcranial direct current stimulation on chronic ankle instability. Med. Sci. Sports Exerc 52, 335–344. 10.1249/MSS.0000000000002129 31453883

[B4] Burcal C. J. Needle A. R. Custer L. Rosen A. B. (2019). The effects of cognitive loading on motor behavior in injured individuals: a systematic review. Sports Med. 49, 1233–1253. 10.1007/s40279-019-01116-7 31066022

[B5] Bush G. Luu P. Posner M. I. (2000). Cognitive and emotional influences in anterior cingulate cortex. Trends Cogn. Sci. 4, 215–222. 10.1016/s1364-6613(00)01483-2 10827444

[B6] Choi J. Y. Yoo T. Burcal C. J. Rosen A. B. (2023). Dual-task differences in individuals with chronic ankle instability: a systematic review with meta-analysis. Gait Posture 106, 28–33. 10.1016/j.gaitpost.2023.08.013 37639962

[B7] Cohen J. Cohen J. Cohen J. W. Cohen J. Cohen J. Cohen J. (1988). Statistical power analysis for the behavioral sciences. Technometrics 31, 499–500. 10.2307/1270020

[B8] Di Giminiani R. Jozsef T. Francesco M. (2020). Gender differences on neuromuscular strategy during drop jump: a comment on Helm et al. (2019). Eur. J. Appl. Physiol. 120, 2555–2556. 10.1007/s00421-020-04465-8 32772247

[B9] Doherty C. Delahunt E. Caulfield B. Hertel J. Ryan J. Bleakley C. (2014). The incidence and prevalence of ankle sprain injury: a systematic review and meta-analysis of prospective epidemiological studies. Sports Med. 44, 123–140. 10.1007/s40279-013-0102-5 24105612

[B10] Dong S. Liu Y. Liu Z. Shen P. Sun H. Zhang P. (2024). Can arthrogenic muscle inhibition exist in peroneal muscles among people with chronic ankle instability? A cross-sectional study. Sports Med. Open 10, 35. 10.1186/s40798-024-00710-y 38598018 PMC11006644

[B11] Fong D. T. Ha S. C. Mok K. M. Chan C. W. Chan K. M. (2012). Kinematics analysis of ankle inversion ligamentous sprain injuries in sports: five cases from televised tennis competitions. Am. J. Sports Med. 40, 2627–2632. 10.1177/0363546512458259 22967824

[B12] Fong D. T. Hong Y. Chan L. K. Yung P. S. Chan K. M. (2007). A systematic review on ankle injury and ankle sprain in sports. Sports Med. 37, 73–94. 10.2165/00007256-200737010-00006 17190537

[B13] Fong D. T. H. Y. Shima Y. Krosshaug T. Yung P. S. Chan K. M. (2009). Biomechanics of supination ankle sprain: a case report of an accidental injury event in the laboratory. Am. J. Sports Med. 37 (4), 822–827. 10.1177/0363546508328102 19188559

[B14] Ford K. R. Myer G. D. Hewett T. E. (2007). Reliability of landing 3D motion analysis: implications for longitudinal analyses. Med. Sci. Sports Exerc 39, 2021–2028. 10.1249/mss.0b013e318149332d 17986911

[B15] Fu W. Fang Y. Liu Y. Hou J. (2014). The effect of high-top and low-top shoes on ankle inversion kinematics and muscle activation in landing on a tilted surface. J. Foot Ankle Res. 7, 14. 10.1186/1757-1146-7-14 24548559 PMC3943374

[B16] Gribble P. A. (2019). Evaluating and differentiating ankle instability. J. Athl. Train. 54, 617–627. 10.4085/1062-6050-484-17 31161943 PMC6602389

[B17] Gribble P. A. Bleakley C. M. Caulfield B. M. Docherty C. L. Fourchet F. Fong D. T. (2016a). 2016 consensus statement of the International Ankle Consortium: prevalence, impact and long-term consequences of lateral ankle sprains. Br. J. Sports Med. 50, 1493–1495. 10.1136/bjsports-2016-096188 27259750

[B18] Gribble P. A. Bleakley C. M. Caulfield B. M. Docherty C. L. Fourchet F. Fong D. T. (2016b). Evidence review for the 2016 International Ankle Consortium consensus statement on the prevalence, impact and long-term consequences of lateral ankle sprains. Br. J. Sports Med. 50, 1496–1505. 10.1136/bjsports-2016-096189 27259753

[B19] Herzog M. M. Kerr Z. Y. Marshall S. W. Wikstrom E. A. (2019). Epidemiology of ankle sprains and chronic ankle instability. J. Athl. Train. 54, 603–610. 10.4085/1062-6050-447-17 31135209 PMC6602402

[B20] Hiller C. E. Refshauge K. M. Bundy A. C. Herbert R. D. Kilbreath S. L. (2006). The Cumberland ankle instability tool: a report of validity and reliability testing. Arch. Phys. Med. Rehabil. 87, 1235–1241. 10.1016/j.apmr.2006.05.022 16935061

[B21] Huang X. Gao H. Fu H. (2024). Effects of transcranial direct current stimulation combined with Bosu ball training on the injury potential during drop landing in people with chronic ankle instability. Front. Physiol. 15, 1451556. 10.3389/fphys.2024.1451556 39210968 PMC11359566

[B22] Hubbard-Turner T. Turner M. J. (2015). Physical activity levels in college students with chronic ankle instability. J. Athl. Train. 50, 742–747. 10.4085/1062-6050-50.3.05 25898110 PMC4532186

[B23] Kemler E. Van De Port I. Valkenberg H. Hoes A. W. Backx F. J. (2015). Ankle injuries in The Netherlands: trends over 10-25 years. Scand. J. Med. Sci. Sports 25, 331–337. 10.1111/sms.12248 24840653

[B24] Konradsen L. Bech L. Ehrenbjerg M. Nickelsen T. (2002). Seven years follow-up after ankle inversion trauma. Scand. J. Med. Sci. Sports 12, 129–135. 10.1034/j.1600-0838.2002.02104.x 12135444

[B25] Koshino Y. Ishida T. Yamanaka M. Samukawa M. Kobayashi T. Tohyama H. (2017). Toe-in landing increases the ankle inversion angle and moment during single-leg landing: implications in the prevention of lateral ankle sprains. J. Sport Rehabil. 26, 530–535. 10.1123/jsr.2016-0004 27992246

[B26] Kosik K. B. Terada M. Drinkard C. P. Mccann R. S. Gribble P. A. (2017). Potential corticomotor plasticity in those with and without chronic ankle instability. Med. Sci. Sports Exerc 49, 141–149. 10.1249/MSS.0000000000001066 27501358

[B27] Lacour M. Bernard-Demanze L. Dumitrescu M. (2008). Posture control, aging, and attention resources: models and posture-analysis methods. Neurophysiol. Clin. 38, 411–421. 10.1016/j.neucli.2008.09.005 19026961

[B28] Lim Y. Y. Sterzing T. Teo C. J. Y. Alonzo R. Pan J. W. Teng P. S. P. (2020). Between-limb asymmetry in kinetic and temporal characteristics during bilateral plyometric drop jumps from different heights. J. Sports Sci. 38, 1605–1614. 10.1080/02640414.2020.1752535 32286154

[B29] Li Y. Ko J. Walker M. A. Brown C. N. Schmidt J. D. Kim S. H. (2018). Does chronic ankle instability influence lower extremity muscle activation of females during landing? J. Electromyogr. Kinesiol 38, 81–87. 10.1016/j.jelekin.2017.11.009 29175719

[B30] Mackala K. Rauter S. Simenko J. Kreft R. Stodolka J. Krizaj J. (2020). The effect of height on drop jumps in relation to somatic parameters and landing kinetics. Int. J. Environ. Res. Public Health 17, 5886. 10.3390/ijerph17165886 32823725 PMC7459744

[B31] Mccahill J. Stebbins J. Theologis T. (2008). Use of the Oxford foot model in clinical practice. J. Foot Ankle Res. 1, O28. 10.1186/1757-1146-1-s1-o28

[B32] Mcleod M. M. Gribble P. A. Pietrosimone B. G. (2015). Chronic ankle instability and neural excitability of the lower extremity. J. Athl. Train. 50, 847–853. 10.4085/1062-6050-50.4.06 26090710 PMC4629942

[B33] Mcnevin N. H. Wulf G. (2002). Attentional focus on supra-postural tasks affects postural control. Hum. Mov. Sci. 21, 187–202. 10.1016/s0167-9457(02)00095-7 12167298

[B34] Medina Mckeon J. M. Hoch M. C. (2019). The ankle-joint complex: a kinesiologic approach to lateral ankle sprains. J. Athl. Train. 54, 589–602. 10.4085/1062-6050-472-17 31184957 PMC6602390

[B35] Menacho Mde O. Pereira H. M. Oliveira B. I. Chagas L. M. Toyohara M. T. Cardoso J. R. (2010). The peroneus reaction time during sudden inversion test: systematic review. J. Electromyogr. Kinesiol 20, 559–565. 10.1016/j.jelekin.2009.11.007 20083415

[B36] Mokhtarzadeh H. Yeow C. H. Goh J. C. H. Oetomo D. Ewing K. Lee P. V. S. (2017). Antagonist muscle co-contraction during a double-leg landing maneuver at two heights. Comput. Methods Biomech. Biomed. Engin 20, 1382–1393. 10.1080/10255842.2017.1366992 28836455

[B37] Nanbancha A. Tretriluxana J. Limroongreungrat W. Sinsurin K. (2019). Decreased supraspinal control and neuromuscular function controlling the ankle joint in athletes with chronic ankle instability. Eur. J. Appl. Physiol. 119, 2041–2052. 10.1007/s00421-019-04191-w 31321512

[B38] Needle A. R. Lepley A. S. Grooms D. R. (2017). Central nervous system adaptation after ligamentous injury: a summary of theories, evidence, and clinical interpretation. Sports Med. 47, 1271–1288. 10.1007/s40279-016-0666-y 28005191

[B39] Norte G. Rush J. Sherman D. (2022). Arthrogenic muscle inhibition: best evidence, mechanisms, and theory for treating the unseen in clinical rehabilitation. J. Sport Rehabil. 31, 717–735. 10.1123/jsr.2021-0139 34883466

[B40] Oh M. Lee H. Han S. Hopkins J. T. (2024). Postural control measured before and after simulated ankle inversion landings among individuals with chronic ankle instability, copers, and controls. Gait Posture 107, 17–22. 10.1016/j.gaitpost.2023.09.002 37716278

[B41] Pierce C. A. Block R. A. Aguinis H. (2004). Cautionary note on reporting eta-squared values from multifactor ANOVA designs. Educ. Psychol. Meas. 64, 916–924. 10.1177/0013164404264848

[B42] Pietrosimone B. G. Gribble P. A. (2012). Chronic ankle instability and corticomotor excitability of the fibularis longus muscle. J. Athl. Train. 47, 621–626. 10.4085/1062-6050-47.6.11 23182009 PMC3499885

[B43] Pietrosimone B. G. Mcleod M. M. Lepley A. S. (2012). A theoretical framework for understanding neuromuscular response to lower extremity joint injury. Sports Health 4, 31–35. 10.1177/1941738111428251 23016066 PMC3435894

[B44] Rahnama L. Salavati M. Akhbari B. Mazaheri M. (2010). Attentional demands and postural control in athletes with and without functional ankle instability. J. Orthop. Sports Phys. Ther. 40, 180–187. 10.2519/jospt.2010.3188 20195021

[B45] Rigby R. Cottom J. M. Rozin R. (2015). Isolated calcaneofibular ligament injury: a report of two cases. J. Foot Ankle Surg. 54, 487–489. 10.1053/j.jfas.2014.08.017 25441852

[B46] Rosen A. B. Mcgrath M. L. Maerlender A. L. (2021). Males with chronic ankle instability demonstrate deficits in neurocognitive function compared to control and copers. Res. Sports Med. 29, 116–128. 10.1080/15438627.2020.1723099 31992081 PMC7387149

[B47] Shen Y. Wang W. Wang Y. Yang L. Yuan C. Yang Y. (2022). Not only in sensorimotor network: local and distant cerebral inherent activity of chronic ankle instability-A resting-state fMRI study. Front. Neurosci. 16, 835538. 10.3389/fnins.2022.835538 35197822 PMC8859266

[B48] Shibata S. Takemura M. Miyakawa S. (2023). Kinematics, kinetics and muscle activity analysis during single-leg drop-jump landing followed by an unanticipated task: focusing on differences in neurocognitive function. Int. J. Sports Phys. Ther. 18, 1085–1093. 10.26603/001c.86124 37795316 PMC10547070

[B49] Shiravi Z. Talebian Moghadam S. Hadian M. R. Olyaei G. (2017). Effect of cognitive task on postural control of the patients with chronic ankle instability during single and double leg standing. J. Bodyw. Mov. Ther. 21, 58–62. 10.1016/j.jbmt.2016.05.001 28167191

[B50] Simpson J. D. Koldenhoven R. M. Wilson S. J. Stewart E. M. Turner A. J. Chander H. (2022). Lower extremity joint kinematics of a simulated lateral ankle sprain after drop landings in participants with chronic ankle instability. Sports Biomech. 21, 428–446. 10.1080/14763141.2021.1908414 33896373

[B51] Stearns-Reider K. M. Powers C. M. (2018). Rate of torque development and feedforward control of the hip and knee extensors: gender differences. J. Mot. Behav. 50, 321–329. 10.1080/00222895.2017.1363692 28985154

[B52] Tavakoli S. Forghany S. Nester C. (2016). The effect of dual tasking on foot kinematics in people with functional ankle instability. Gait Posture 49, 364–370. 10.1016/j.gaitpost.2016.07.302 27494304

[B53] Terada M. Kosik K. B. Mccann R. S. Drinkard C. Gribble P. A. (2022). Corticospinal activity during a single-leg stance in people with chronic ankle instability. J. Sport Health Sci. 11, 58–66. 10.1016/j.jshs.2020.08.008 32866712 PMC8847849

[B54] Terrier R. Rose-Dulcina K. Toschi B. Forestier N. (2014). Impaired control of weight bearing ankle inversion in subjects with chronic ankle instability. Clin. Biomech. (Bristol, Avon) 29, 439–443. 10.1016/j.clinbiomech.2014.01.005 24485883

[B55] Vuillerme N. Nougier V. Teasdale N. (2000). Effects of a reaction time task on postural control in humans. Neurosci. Lett. 291, 77–80. 10.1016/s0304-3940(00)01374-4 10978578

[B56] Watanabe K. Koshino Y. Ishida T. Samukawa M. Tohyama H. (2022). Energy dissipation during single-leg landing from three heights in individuals with and without chronic ankle instability. Sports Biomech. 21, 408–427. 10.1080/14763141.2021.2009549 34872455

[B57] Wrightson J. G. SchäFER L. Smeeton N. J. (2020). Dual-task prioritization during overground and treadmill walking in healthy adults. Gait Posture 75, 109–114. 10.1016/j.gaitpost.2019.08.007 31669806

[B58] Wu G. Siegler S. Allard P. Kirtley C. Leardini A. Rosenbaum D. (2002). ISB recommendation on definitions of joint coordinate system of various joints for the reporting of human joint motion--part I: ankle, hip, and spine. International Society of Biomechanics. J. Biomech. 35, 543–548. 10.1016/s0021-9290(01)00222-6 11934426

[B59] Wulf G. Mercer J. Mcnevin N. Guadagnoli M. A. (2004). Reciprocal influences of attentional focus on postural and suprapostural task performance. J. Mot. Behav. 36, 189–199. 10.3200/JMBR.36.2.189-199 15130869

[B60] Xu D. Zhou H. Quan W. Gusztav F. Wang M. Baker J. S. (2023). Accurately and effectively predict the ACL force: utilizing biomechanical landing pattern before and after-fatigue. Comput. Methods Programs Biomed. 241, 107761. 10.1016/j.cmpb.2023.107761 37579552

[B61] Xu D. Zhou H. Quan W. Ma X. Chon T. E. Fernandez J. (2024a). New insights optimize landing strategies to reduce lower limb injury risk. Cyborg Bionic Syst. 5, 0126. 10.34133/cbsystems.0126 38778877 PMC11109754

[B62] Xu D. Zhou H. Wang M. Ma X. Gusztav F. Chon T. E. (2024b). Contribution of ankle motion pattern during landing to reduce the knee-related injury risk. Comput. Biol. Med. 180, 108965. 10.1016/j.compbiomed.2024.108965 39084051

[B63] YıLDıZ S. YalcıN B. (2013). The anterior talofibular and calcaneofibular ligaments: an anatomic study. Surg. Radiol. Anat. 35, 511–516. 10.1007/s00276-012-1071-3 23292089

[B64] Zhang T. Zhu X. Li L. Zhou Z. Shen P. Fong D. T. P. (2024). Different strategies for landing from different heights among people with chronic ankle instability. Gait Posture 114, 90–94. 10.1016/j.gaitpost.2024.09.008 39293282

